# Supplementary material for: The causal relationship between the human gut microbiota and pyogenic arthritis: a Mendelian randomization study
Source: Front Cell Infect Microbiol. 2024 Nov 26;14:1452480. doi: 10.3389/fcimb.2024.1452480 (PMC11629706; doi:10.3389/fcimb.2024.1452480)
Supplement: Supplementary file 1 [file DataSheet1.zip › mendelian test/ebi-a-GCST90027697.csv_leave_one_out.pdf]

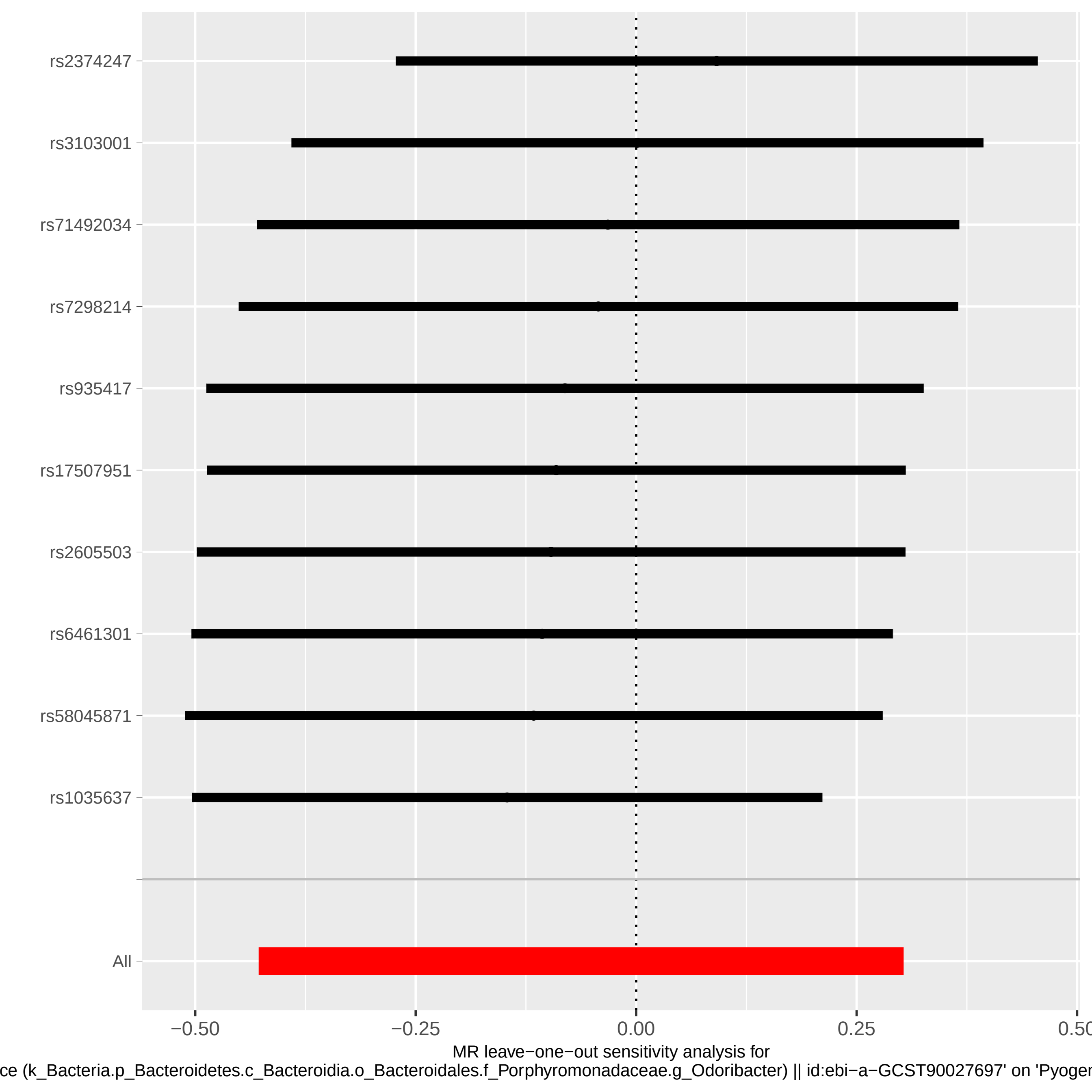
rs2374247
rs3103001
rs71492034
rs7298214
rs935417
rs17507951
rs2605503
rs6461301
rs58045871
rs1035637
All
−0.50
−0.25
0.00
0.25
0.50
MR leave−one−out sensitivity analysis for
ce (k_Bacteria.p_Bacteroidetes.c_Bacteroidia.o_Bacteroidales.f_Porphyromonadaceae.g_Odoribacter) || id:ebi−a−GCST90027697' on 'Pyoger
